# Are bacteria potential sources of fish environmental DNA?

**DOI:** 10.1371/journal.pone.0230174

**Published:** 2020-03-12

**Authors:** Kei Nukazawa, Kentaro Akahoshi, Yoshihiro Suzuki

**Affiliations:** Department of Civil and Environmental Engineering, Faculty of Engineering, University of Miyazaki, Miyazaki, Japan; University of Hyogo, JAPAN

## Abstract

The environmental DNA (eDNA) method is being increasingly applied in various environments. Although eDNA undergoes rapid degradation in aqueous environments, it has been detected in streams up to 10 km downstream from its source. As environmental bacteria can uptake free DNA, transfer their genetic traits, and amplify, there is a potential risk that they, rather than a target aquatic species, could become a source of measured eDNA. This study examined whether bacteria with incorporated fish DNA could be such a source by investigating the detectability of fish DNA generated by bacteria inhabiting river water and riverbed sediment. We attempted to detect common carp (*Cyprinus carpio*) eDNA in stream water and sediment samples and the DNA of common carp produced by bacterial colonies (*Escherichia coli*, total coliform, and heterotrophic bacteria) cultured from the samples. The eDNA was detected in the environmental samples but the carp DNA from the targeted bacteria was rarely detected in both water and riverbed sediment samples. Our results suggest that the risk of bacterium-induced false positive detection for fish eDNA is negligible.

## 1. Introduction

The environmental DNA (eDNA) method has been increasingly applied to various organisms and environments [1–5]. Although many eDNA studies have focused on detecting the presence or absence of species, the eDNA technique also shows considerable potential for inferring species abundance in lotic and lentic systems based on quantified copy numbers of target DNA fragments [6]. Despite the potential advantages of the method, considerable challenges remain in terms of understanding the fate and dynamics of eDNA in water bodies. eDNA is genetic material that presents in environments such as water and soil originated from excreted cells or tissue including saliva, feces, urine [1,7]. It has been shown that stream eDNA can be degraded by physical processes (e.g., advection and settling) as well as chemical and biological processes (e.g., hydrolysis by nucleases)[8]. The physical settling and subsequent resuspension of eDNA from a target taxon creates the risk of false positive detection. Consequently, even if a target taxon is detected with the eDNA technique, the distribution of that taxon remains unclear. Hence, a better understanding of stream eDNA dynamics is required for more reliable biological monitoring.

Previous research has investigated the transport distance of eDNA in rivers; the eDNA of juvenile salamanders (*Dicamptodon aterrimus*) could not be detected 50 m downstream of their source [9], whereas those of common carp (*Cyprinus carpio*; approx. 100 farmed adult individuals) and invertebrates (inhabiting a natural lake) could be detected 3 km and 10 km downstream [8,10]. These contradictory results may be ascribed to a contrast in the abundance of the source species [8]. However, as eDNA degrades fairly rapidly in streams, its transport over such relatively long distances (≥ 3 km) must be explained. Fish eDNA has been reported in higher concentrations in riverbed sediment than in water bodies [11], even though microbes that can degrade eDNA [12–14] are also present in higher concentrations in riverbed substrate. Whereas extraceller DNA absorbed on aquatic sediment particles could be protected from degradation and experience long-term persistence [15,16], another potential pathway of such DNA could be transformation onto environmental bacteria through horizontal gene transfer. Natural bacteria are known to uptake free DNA and transfer its genetic traits [17]. Thus, if bacteria in water bodies and riverbed substrate uptake and amplify eDNA, they could be an additional source of eDNA that interferes with accurate and precise monitoring of target species.

This study demonstrates bacterial uptake of eDNA by investigating the detectability of carp DNA from bacteria inhabiting river water and bed sediment. We sampled stream water and sediment and attempted to detect common carp DNA in the environmental samples and from cultured bacterial groups (*Escherichia coli*, total coliforms, and heterotrophic bacteria). *E*. *coli* and total coliforms were chosen because they are widely distributed major bacterial groups that would be suitable to track transformation in aquatic environments. Also, these groups have shown great potential of horizontal gene transfer such as acquisition of antibiotic resistant genes [18,19]. Heterotrophic bacteria were studied because heterotrophic traits might be essential for effective bacterial uptake and transformation of eDNA.

## 2. Materials and methods

### 2.1. Stream water and sediment samples

Samples were collected from the Kaeda River in southwest Japan. Effluent from a common carp farm enters the river at its uppermost reach. In a previous study, carp eDNA was detected in the river downstream of the farm [8]. In the present study, we sampled downstream river water and riverbed sediment as a positive sample of the carp eDNA in May 2018. On the same date, we also sampled river water and riverbed sediment upstream of the farm (approx. 800 m) as a negative control. For a more detailed description of the river studied, see [7].

Stream water samples were collected in triplicate at the downstream site in 5 L plastic bottles before the riverbed sediment was sampled. Sediment samples on the riverbed surface were composed mainly of sand; they were collected at the downstream site, in triplicate, with a vinyl chloride pipe and transported to the laboratory in plastic bags. Single samples of water and sediment were also collected at the upstream site. The samples were transported on ice in cooler boxes and underwent filtration within 4 h. Prior to sampling, the bottles and cooler boxes were sterilized with 10% bleach for at least 30 min.

**Fig 1:**
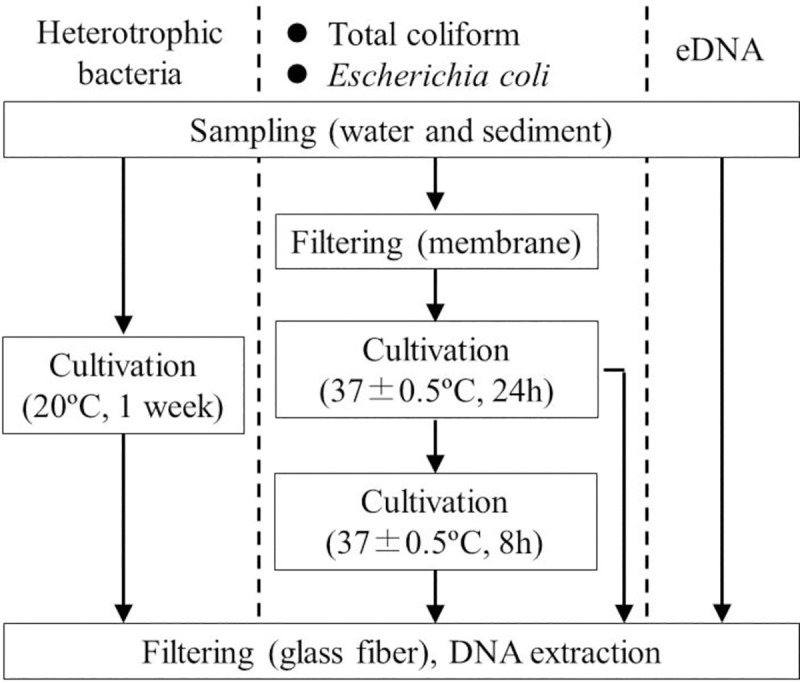
Flowchart of experimental procedures used to detect carp DNA from cultivated bacteria.

The samples were used to test for the presence of common carp eDNA, following the usual protocol [8] and cultivate bacterial groups and test for the presence of common carp DNA in the isolated strains. The experimental procedures are summarized in Fig 1 and detailed in sections 2.2–2.5.

### 2.2. Filtration

One liter of water was separated from the 5 L samples and filtered using a glass fiber filter with a pore size of 0.7 μm (GE Healthcare Japan, Tokyo). To acquire sufficient colonies to detect carp DNA from *E*. *coli* and total coliforms, three 500 mL aliquots of water were filtered from each replicate, using a membrane filter with a pore size of 0.45 μm (Advantec, Japan). In addition, 50 mL of water was filtered from each sample to enumerate the *E*. *coli* and total coliform colonies.

For each sediment sample, 5 g of sediment was transferred to a 50 mL centrifuge tube containing 40 mL physiological saline solution and mixed intensively for 2 min. The mixed solution was allowed to settle for 1 min before the supernatant was filtered through glass fiber filters to detect eDNA and through membrane filters to isolate and enumerate the target bacteria, using the same procedures as for the water samples. Filter funnels, bases, clamps, and tweezers were sterilized in 10% bleach for 10 min prior to each filtration process. The glass fiber filters were then stored in a freezer at -20°C until use in DNA extraction.

### 2.3. Cultivation, enumeration, and isolation of target bacteria

To cultivate *E*. *coli* and total coliforms, the filtered membranes were set on CHROMagar^TM^ ECC media (a selective medium for both *E*. *coli* and total coliform; CHROMagar^TM^, Paris, France), and cultured at 37 ± 0.5°C for 24 h. After cultivation, red-violet and blue colonies were identified as total coliform and *E*. *coli*, respectively, and mean concentrations were calculated from the three replicates as background information of target bacterial abundance. Only two replicates of total coliform isolated from water samples were available at the downstream site. One hundred cultivated colonies for each bacterial taxon and sample were randomly isolated using sterilized toothpicks and suspended in brain heart infusion liquid medium. The partially suspended samples were subsequently filtered using glass fiber filters. The other samples collected at the downstream site were enriched by cultivation at 37 ± 0.5°C for an additional 8 h. These samples were also filtered using glass fiber filters. Bacteria from samples collected from the upstream site were not enriched.

Heterotrophic bacteria were cultivated by applying the water samples directly to R2A agar media and incubating under sterile conditions at 20°C for 1 week. Subsequently, the cultured strains were aggregated using a bacteria spreader and suspended in 1 mL physiological saline solution. The suspended samples were then filtered using glass fiber filters.

### 2.4. DNA extraction

The DNA was extracted from processed glass fiber filters using a DNeasy PowerSoil Kit (Qiagen, Hilden, Germany), which shows a lower variation in eDNA quantification for common carp than other extraction methods and has no detectable inhibition [20]. Sterilized tweezers and scissors were used to cut the filters into 1 × 3 mm pieces. These small filter fragments were used for DNA extraction following the manufacturer instructions. The double-strand DNA (dsDNA) concentration for each sample was measured with a fluorometer (Quantus, Promega, WI, USA). The extracted template DNA solutions were stored in a freezer at -20°C until they were used in the polymerase chain reaction (PCR) analysis.

### 2.5. Detection of common carp DNA using digital polymerase chain reaction (PCR)

To detect the DNA of common carp, we used the primer and probe set to target mitochondrial cytochrome b specific to common carp [21]. The specificity of the assay had been validated for the study area [8]. When compared with quantitative polymerase chain reaction systems, dPCR produces a more stable quantification given low concentrations of common carp eDNA [22]. Each reaction mixture for DNA quantification contained 1 × QuantStudio 3D Digital PCR Master Mix (Applied Biosystems, CA, USA), 2 μL of DNA template solution, 900 nM of each primer (forward and reverse), and 125 nM of TaqMan probe. The mixture was dispensed into independent wells of a QuantStudio 3D Digital PCR 20K Chip with a QuantStudio 3D Digital PCR Chip Loader (Applied Biosystems, CA, USA). The endpoint PCR reaction was performed using a thermal cycler (ProFlex, Applied Biosystems, CA, USA). The PCR reactions followed the default dPCR protocol: polymerase activation at 96°C for 10 min followed by 40 cycles of annealing and extension at 60°C for 2 min, denaturation at 98°C for 30 s, and final extension at 60°C for 2 min. We used the QuantStudio 3D digital PCR system and QuantStudio 3D Analysis Suite software (Applied Biosystems, CA, USA) to detect common carp DNA. To discriminate positive and negative wells from 20,000 wells in dPCR platform, we considered software’s default fluorescence intensity threshold to discriminate positive and negative wells. If the number of positive wells was less than 10 with weak fluorescence intensity and not clearly separated from a group of negative wells, or the number of positive wells was less than 20 with weak fluorescence intensity and not separated from a group of negative wells, the sample was considered negative. If the sample was judged as positive, we quantified the carp DNA concentration in the DNA solution. Furthermore, if the bacterial sample was judged as positive, we performed additional two dPCR experiments to confirm reliability of the results. The dPCR procedure was performed in a different room than that in which the filtration and DNA extraction processes were carried out; none of the instruments were transferred between the rooms.

## 3. Results

**Fig 2:**
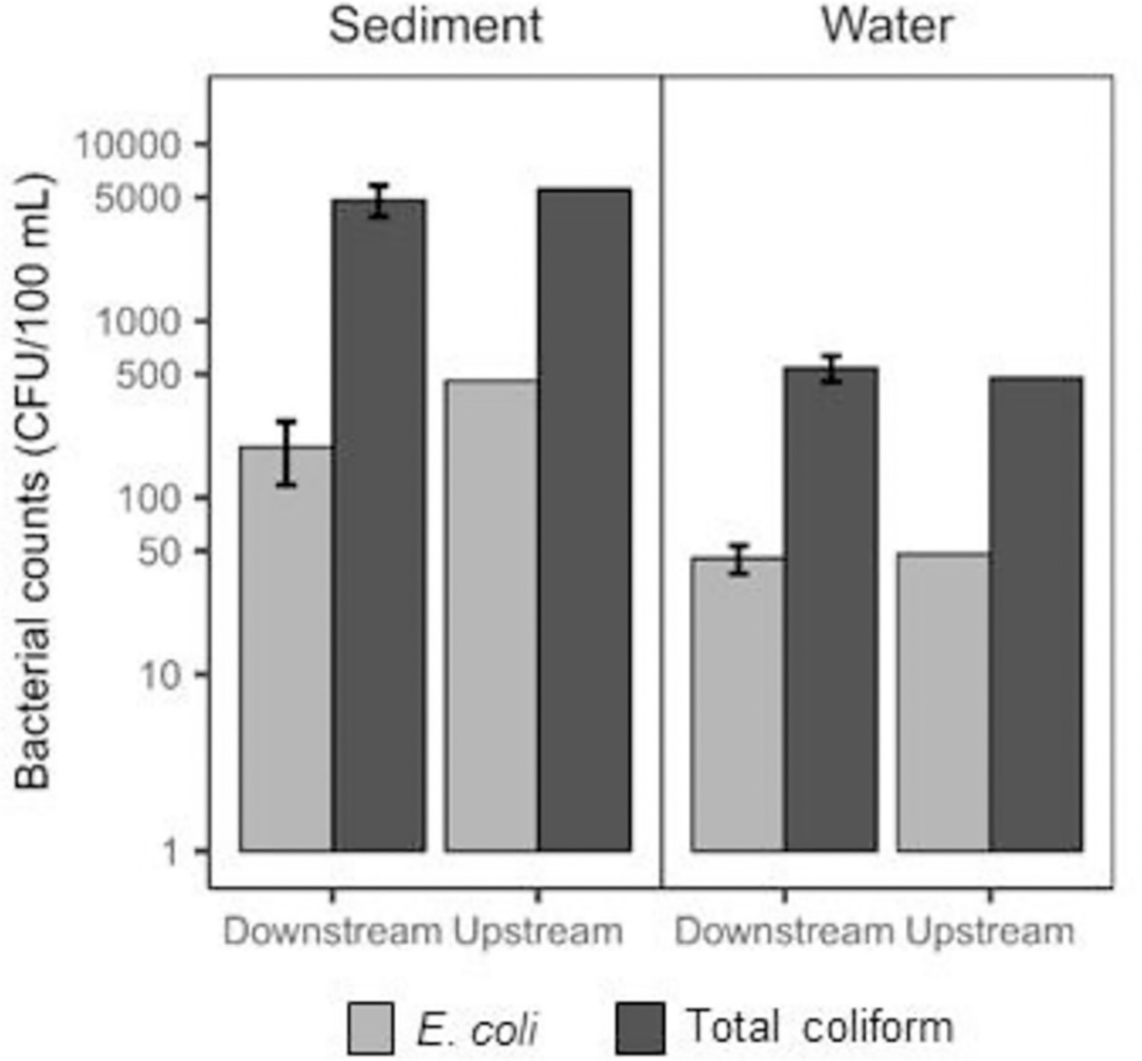
Bacterial concentration in water and sediment samples at the study sites. Error bars show the standard deviation of the triplicate samples.

Fig 2 shows the results of the *E*. *coli* and total coliform quantification. At the downstream site, the concentrations of *E*. *coli* and total coliform from the water sample were 45.33 CFU/100 mL and 543.33 CFU/100 mL, respectively. The concentrations of *E*. *coli* and total coliform from the sediment sample were 193.33 CFU/100 g and 4846.67 CFU/100 g, respectively. In addition, the dsDNA concentrations were generally higher in the sediment samples compared with the water samples at the downstream and upstream site (3.75 and 10.2 μg/100 g vs. 0.0217 and 0.0247 μg/100 mL, respectively). Bacterial abundance was clearly much higher in the riverbed substrate samples than in the stream water samples.

**Fig 3:**
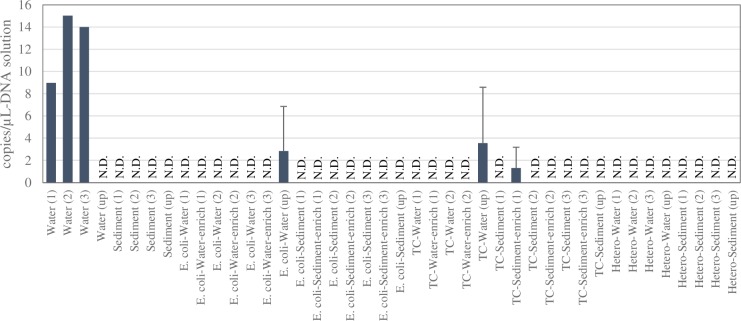
Results of detections of common carp DNA from environmental samples, *E*.*coli*, total coliforms (TC) and heterotrophic bacteria (Hetero) at the downstream (three replicates) and upstream (single replicate) sites. Parenthesized numeric indicates replicate sample at the downstream site while “up” indicates sample at the upstream site. N.D. indicates negative result in dPCR analysis. Two replicates were used for total coliform and enriched total coliform measurements from the water samples.

Fig 3 presents the results of carp DNA detected in the environmental and bacterial samples. There was no common carp eDNA detected in the environmental samples taken from the upstream site. This indicated that eDNA inputs other than the effluent from the carp farm were negligible in the present study. At the downstream site, eDNA was positively detected in triplicate water samples, ranging from 8.965 to 15.028 copies/μL-DNA solution. None of the triplicate sediment samples showed a positive detection for eDNA.

Common carp DNA was detected once in the triplicate tests of *E*. *coli* isolated from the water sample at the upstream site; however, it was not detected in *E*. *coli* isolated from any other samples from the downstream and upstream sites (Fig 3). Furthermore, DNA was not detected in enriched *E*. *coli* cultivated from water or sediment samples from both sites. Common carp DNA was undetected in the total coliform isolated from both the water and sediment samples at the downstream site, but those form water sample was detected at the upstream site. However, for enriched total coliform isolated from the sediment sample, the carp DNA was detected once per three replicates at the downstream site but not at the upstream site. The common carp DNA was undetected in the heterotrophic bacteria isolated from both water and sediment samples at the downstream and upstream sites.

We performed additional two dPCR experiments to verify whether the positive signals of carp DNA observed in *E*. *coli* and total coliforms (three samples) are likely or not. As a result, none of the additional experiments was considered positive; resulting in small carp DNA concentration with large error bar in Fig 3.

## 4. Discussion

In contrast to the eDNA detection results targeting water samples, we rarely observed clear DNA detection patterns for the cultivated bacterial groups. Carp DNA was detected from isolated *E*. *coli* strains from upstream water with lower copy number and detection frequency. If *E*. *coli* strains contained carp DNA, it would likely have been detectable in the enriched strains, but such trend was not observed. In addition, eDNA of common carp was not observed upstream of the carp farm in this study as well as retrospective observations [8], suggesting no carp individuals inhabit in the upstream corridor. These facts contradict the results that retrieve carp DNA from any media upstream. Therefore, it appears that naturalized *E*. *coli* in river did not reproduce common carp DNA and were therefore not a probable source of false positive results. Positive results of carp DNA observed in total coliforms were also considered false due to low concentration of carp DNA with low detection frequency at upstream and only sole enrich sample. While no firm conclusions about the uptake, transformation, and amplification of eDNA for *E*. *coli* and total coliforms in streams can be drawn from the inconsistent results, they did at least suggest that the signals for eDNA transformation and amplification by the studied bacterial groups were extremely limited. Also, we could not find any clear signal of carp DNA from heterotrophic bacterium samples both the downstream and upstream sites, suggesting no transformation and amplification occurs in the culturable heterotrophic bacteria.

We only observed clear positive signal of carp eDNA in the river water sample while those in the sediment sample were all negative. Previous studies reported higher concentrations of fish eDNA in sediment than in bulk water [11] but showed that it was detected less in sediment samples [23]. Even though external source of eDNA was demonstrated as fractional in the present study, extracellular carp DNA provided onto the riverbed sediment downstream of the carp farm could be detected as DNA molecules bound to sediment is protected from further degradation [15,16]. Thus, no signal of carp eDNA within the sediment is potentially explained by abundant unbound DNA, which is more quickly degraded form [23].

To the best of our knowledge, it is the first to disclose the effects of bacterial transfer and amplification of fish DNA on eDNA monitoring in river. The results indicated that the carp DNA was undetectable from most target bacterial samples isolated from water and riverbed sediment. Only three samples (out of 34 samples) first showed positive signals, however, the additional experiments revealed that the positive signals are presumably false. Therefore, we conclude that horizontal gene transfer and amplification of carp DNA in environmental bacteria could be extremely limited and thus false-positive detection of eDNA due to environmental bacteria is negligible.
